# Supplementary figures and images for: Glucocorticoids with low-dose anti-IL1 anakinra rescue in severe non-ICU COVID-19 infection: A cohort study
Source: PLoS One. 2020 Dec 16;15(12):e0243961. doi: 10.1371/journal.pone.0243961 (PMC7743937; doi:10.1371/journal.pone.0243961)

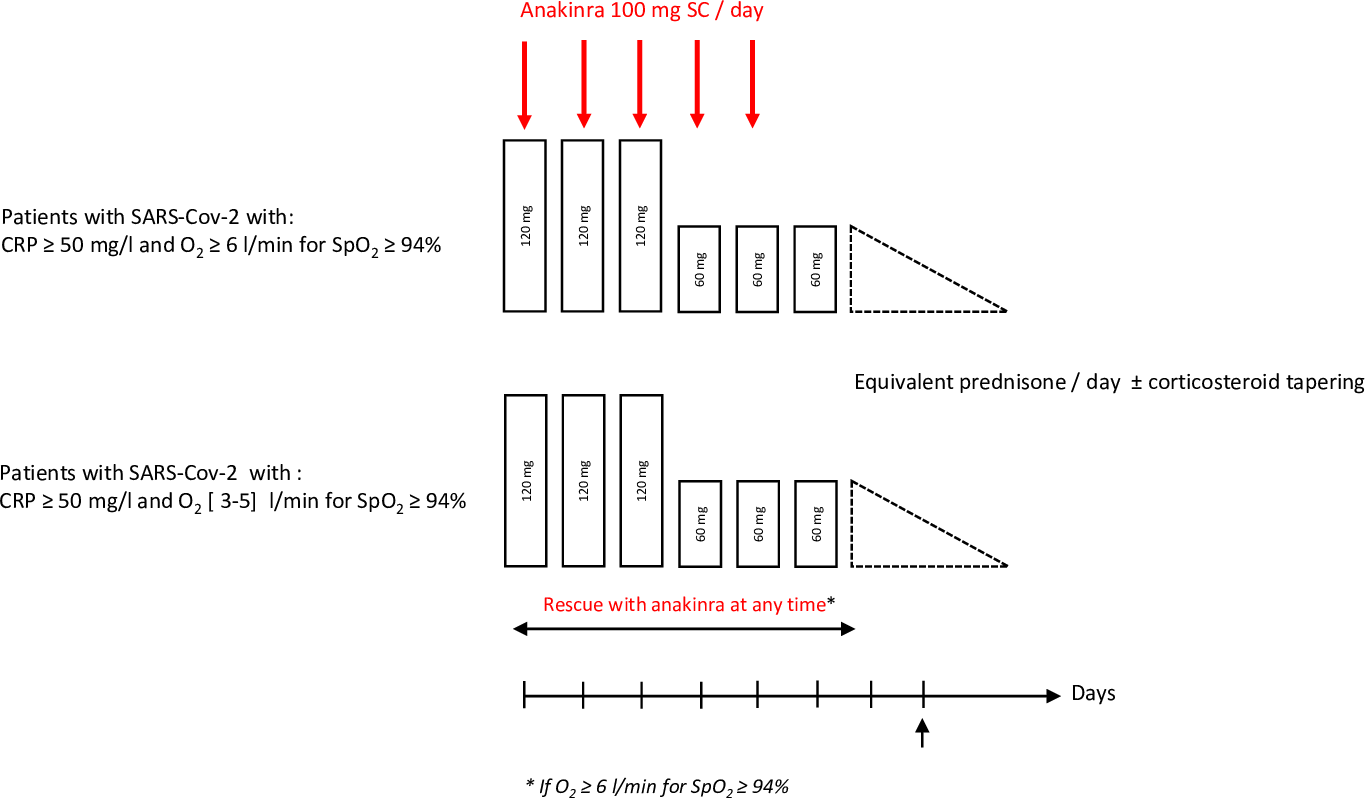

Supplement: S1 Fig — (TIF) [file pone.0243961.s001.tif]

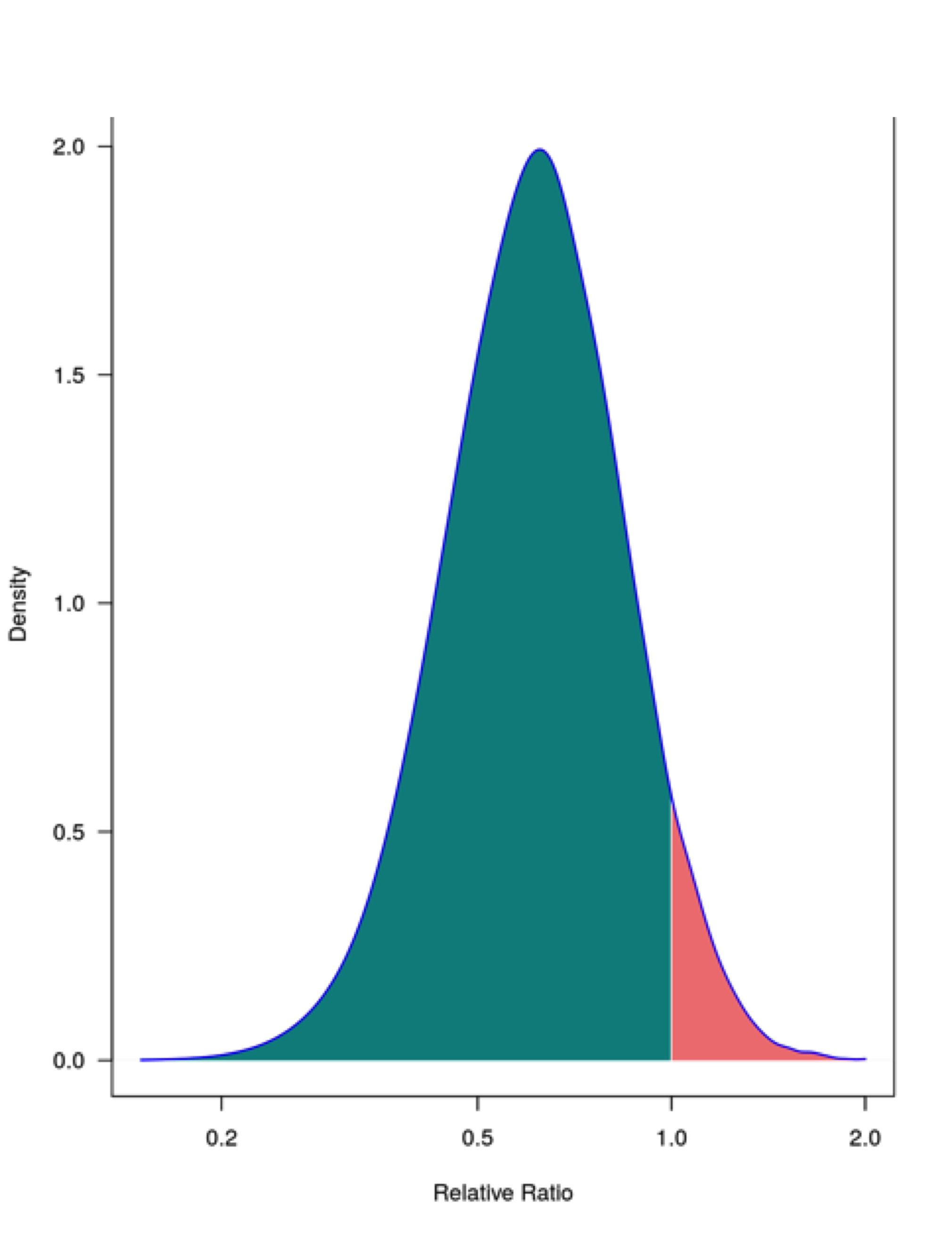

Supplement: S2 Fig — (TIF) [file pone.0243961.s002.tif]
