# Supplementary material for: Glucocorticoids with low-dose anti-IL1 anakinra rescue in severe non-ICU COVID-19 infection: A cohort study
Source: PLoS One. 2020 Dec 16;15(12):e0243961. doi: 10.1371/journal.pone.0243961 (PMC7743937; doi:10.1371/journal.pone.0243961)
Supplement: S1 Table — (DOCX) [file pone.0243961.s003.docx]

**S1 Table: Co-medications in overall population, in the steroids and the control group**

|  | **Overall population**  **(n=171)** | **Steroids-based treatment group**  **(n= 108)** | **Control group**  **(n=63)** | **p-value** |
| --- | --- | --- | --- | --- |
| **Lopinavir/ritonavir**  Yes  No | 62 (36.3%)  109 (63.7%) | 48 (44.4%)  60 (55.6%) | 14 (22.2%)  49 (77.8%) | 0.0035^£^ |
| **ivermectin**  Yes  No | 65/169 (38.5%)  104/169 (61.5%) | 65/106 (61.3%)  41/106 (38.7%) | 0/63 (0.0%)  63/63 (100.0%) | <.0001^¶^ |
| **azithromycin**  Yes  No | 37 (21.6%)  134 (78.4%) | 22 (20.4%)  86 (79.6%) | 15 (23.8%)  48 (76.2%) | 0.60^£^ |
| **long-term previous steroids or other immuno-suppressive drugs**  Yes  No | 14 (8.2%)  157 (91.8%) | 10 (9.3%)  98 (90.7%) | 4 (6.3%)  59 (93.7%) | 0.50^£^ |
| **Hydroxychloroquine**  Yes  No | 20 (11.7%)  151 (88.3%) | 11 (10.2%)  97 (89.8%) | 9 (14.3%)  54 (85.7%) | 0.42^£^ |
| **LMWH**  Yes  No | 156/170^1^ (92.9%)  15/170^1^ (7.1%) | 95/107^1^ (88.8%)  12/107^1^ (11.2%) | 63/63 (100%)  0/63 (0%) | 0.004^¶^ |
| **Remdesivir**  Yes  No | 3/171 (1.8%)  105/171 (98.2%) | 3/108 (2.7%)  105/108 (97.3%) | 0/63 (0%)  63/63 (100%) | Not tested |
| **Tociluzimab**  Yes  No | 3/171 (1.8%)  105/171 (98.2%) | 3/108 (2.7%)  105/108 (97.3%) | 0/63 (0%)  63/63 (100%) | Not tested |

^*^ Two-Sample T-test; ^†^ Mann Whitney U test/Wilcoxon Sum Rank test ; ^¶^ Fisher's exact test; ^£^ Pearson's chi-square test.^&^one patient with no data according heparin
