# Supplementary material for: Glucocorticoids with low-dose anti-IL1 anakinra rescue in severe non-ICU COVID-19 infection: A cohort study
Source: PLoS One. 2020 Dec 16;15(12):e0243961. doi: 10.1371/journal.pone.0243961 (PMC7743937; doi:10.1371/journal.pone.0243961)
Supplement: S2 Table — (DOCX) [file pone.0243961.s004.docx]

**S2 Table: Posterior probabilities of benefit of the treatment, expressed as Odds Ratio (OR), Relative Risk (RR) or Absolute reduction of Risk (ARR) exceeding key landmarks with the different considered priors**.

| Priors | OR<1 | OR<0.9 | OR<0.8 | RR<1 | RR<0.9 | RR<0.8 | ARR >0 | ARR >2 | ARR >10 |
| --- | --- | --- | --- | --- | --- | --- | --- | --- | --- |
| Non-informative | 92.2 | 87.5 | 79.4 | 87.5 | 80.7 | 69.7 | 87.5 | 83.1 | 46.8 |
| Enthusiastic | 98.4 | 96.2 | 91.2 | 97.5 | 94.1 | 86.5 | 97.5 | 95.4 | 64.4 |
| Skeptical | 77.8 | 59.4 | 36.4 | 76.5 | 57.6 | 34.7 | 76.6 | 64.9 | 9.0 |
