# Supplementary material for: Glucocorticoids with low-dose anti-IL1 anakinra rescue in severe non-ICU COVID-19 infection: A cohort study
Source: PLoS One. 2020 Dec 16;15(12):e0243961. doi: 10.1371/journal.pone.0243961 (PMC7743937; doi:10.1371/journal.pone.0243961)
Supplement: S1 File — (DOCX) [file pone.0243961.s005.docx]

**Glucocorticoids with low-dose Anti-IL1 Anakinra Rescue in Severe Non-ICU COVID-19 Infection: a Cohort Study**

# **Supporting information**

# **Supporting Statistical Methods**

A Bayesian analysis allowed estimation of treatment effect on the primary and co-primary outcome as Odds ratio (OR), Risk Ratio (RR) or as Absolute Risk Reduction. (ARR) The posterior probabilities that the treatment effect size exceeded a range of potential values that can be considered as clinically relevant treatment effect were computed for the following values (OR <1, OR <0.9, OR <0.8,RR <1, RR <0.9, RR <0.8, and ARR ≥0%, ARR ≥2%, ARR ≥10 assuming a baseline mortality risk of 33% based on the control group).

Bayesian analysis represents prior beliefs about the plausible range of values for treatment effect as a probability density distribution. A non-informative reference prior, centered at the value of no effect (odds-ratio OR =1) with variance (=10), was used to produce results essentially dependent on data from the trial alone. We also used reference priors that were defined to represent enthusiastic or skeptical, beliefs about the probability of success of the treatment that clinicians may have before the initiation of the study. The enthusiastic prior was centered at the hypothesis that relative risk with the experimental treatment would be around 60% (i.e. corresponding to an OR =0.5 for a death rate of 30% in the control group). The variance for this prior was fixed at 0.42 for which the enthusiastic prior has a small probability (5%) that the steroids treatment is negative. The skeptical prior was centered at the value of no difference with a variance =10. Separate Bayesian models were run for each of the prior distributions on the log OR and log-RR for steroids. The likelihood function (the probability of observing the data collected in the trial for each possible value of OR and RR) was computed for the trial. Each model treated the numbers of deaths in the steroids and control groups as independent samples from binomial distributions and placed a uniform prior on the probability of death in the control group (p_c_) so that the probability in the steroids group was RR × p_c_. Even if there was no unbalance between the two groups regarding baseline characteristics of patients, we used Bayesian adjustment on propensity score to produce estimates of treatment effects ^1^. Markov chain Monte Carlo modeling (with 4 chains) was used to derive treatment effect estimates and 95% credible intervals (CrIs) from the median, 2.5th and 97.5th percentiles of the posterior distribution, and to estimate the posterior probabilities of treatment effects exceeding certain thresholds. The ARR was calculated from the RR for a fixed baseline mortality risk of 33%. The Gelman-Rubin statistic was used to assess the convergence of all models. All analyses were conducted in R (R Foundation) using rstanarm tool (Jonah Gabry et al.): Bayesian applied regression modeling via Stan.

## **Results of Bayesian analysis**

Posterior probabilities of corticoids effect on death with different priors.

Using an enthusiastic prior, the estimates adjusted with propensity score and their 95% Crl were 0.57 (95% CrI, 0.32–0.94) for OR, 0.63 (95% CrI, 0.37–1) for RR, and −0.18 (−0.31, −0.05) for ARR. The posterior probability of an RR of ≤1 or 0.9 exceeded 94% across the enthusiastic prior (eTable 2,) Considering the skeptical prior, the estimated means and 95% Crl were 0.88 (95% CrI: 0.57–1.29) for OR, 0.89 (95% CrI: 0.60–1.29) for RR, and −0.07 (95% CrI: −0.19, 0.05) for ARR. The posterior probability of an RR ≤1 or 0.9 was 77% and 58%, respectively. Even in the skeptical prior case, the posterior probability of an absolute risk reduction that was larger than 2% was 65%. The posterior probabilities of harm (OR >1) were 1.7% and 22.1% for the enthusiastic and skeptical priors, respectively.

# **Reference**

**1**. L. McCandless, P. Gustafson, P. Austin, Bayesian propensity score analysis for observational data *Statistics in Medicine* **28**, 94-112 (2009).
